# An Illustrated Guide to the Imaging Evolution of COVID in Non-Epidemic Areas of Southeast China

**DOI:** 10.3389/fmolb.2021.648180

**Published:** 2021-05-28

**Authors:** Lihua Wang, Yeerfan Jiaerken, Qian Li, Peiyu Huang, Zhujing Shen, Tongtong Zhao, Hanpeng Zheng, Wenbin Ji, Yuantong Gao, Junli Xia, Jianmin Cheng, Jianbing Ma, Jun Liu, Yongqiang Liu, Miaoguang Su, Guixiang Ruan, Jiner Shu, Dawei Ren, Zhenhua Zhao, Weigen Yao, Yunjun Yang, Minming Zhang

**Affiliations:** ^1^ Department of Radiology, Second Affiliated Hospital, School of Medicine, Zhejiang University, Hangzhou, China; ^2^ Fuyang Second People’s Hospital, Fuyang, China; ^3^ Yueqing People's Hospital, Yueqing, China; ^4^ Zhejiang Taizhou Hospital, Taizhou, China; ^5^ Radiology Department, Third Affiliated Hospital of Wenzhou Medical University, Wenzhou, China; ^6^ Bozhou Bone Trauma Hospital Image Center, Bozhou, China; ^7^ Department of Radiology, Second Affiliated Hospital and Yuying Children’s Hospital of Wenzhou Medical University, Wenzhou, China; ^8^ First Hospital of Jiaxing, Jiaxing, China; ^9^ Second Xiangya Hospital, Central South University, Changsha, China; ^10^ Quzhou Kecheng People’s Hospital, Quzhou, China; ^11^ Pingyang County People’s Hospital, Wenzhou, China; ^12^ Yuhang First People’s Hospital, Hangzhou, China; ^13^ Jinhua Central Hospital, Jinhua, China; ^14^ Ningbo First Hospital, Ningbo, China; ^15^ Shaoxing People’s Hospital, Shaoxing, China; ^16^ Yuyao People’s Hospital, Yuyao, China; ^17^ Radiology Department, First Affiliated Hospital of Wenzhou Medical University, Wenzhou, China

**Keywords:** COVID, image, CT, evolution, multicenter

## Abstract

**Purpose:** By analyzing the CT manifestations and evolution of COVID in non-epidemic areas of southeast China, analyzing the developmental abnormalities and accompanying signs in the early and late stages of the disease, providing imaging evidence for clinical diagnosis and identification, and assisting in judging disease progression and monitoring prognosis.

**Methods:** This retrospective and multicenter study included 1,648 chest CT examinations from 693 patients with laboratory-confirmed COVID-19 infection from 16 hospitals of southeast China between January 19 and March 27, 2020. Six trained radiologists analyzed and recorded the distribution and location of the lesions in the CT images of these patients. The accompanying signs include crazy-paving sign, bronchial wall thickening, microvascular thickening, bronchogram sign, fibrous lesions, halo and reverse-halo signs, nodules, atelectasis, and pleural effusion, and at the same time, they analyze the evolution of the abovementioned manifestations over time.

**Result:** There were 1,500 positive findings in 1,648 CT examinations of 693 patients; the average age of the patients was 46 years, including 13 children; the proportion of women was 49%. Early CT manifestations are single or multiple nodular, patchy, or flaky ground-glass–like density shadows. The frequency of occurrence of ground-glass shadows (47.27%), fibrous lesions (42.60%), and microvascular thickening (40.60%) was significantly higher than that of other signs. Ground-glass shadows increase and expand 3–7 days after the onset of symptoms. The distribution and location of lesions were not significantly related to the appearance time. Ground-glass shadow is the most common lesion, with an average absorption time of 6.2 days, followed by consolidation, with an absorption time of about 6.3 days. It takes about 8 days for pure ground-glass lesions to absorb. Consolidation change into ground glass or pure ground glass takes 10–14 days. For ground-glass opacity to evolve into pure ground-glass lesions, it takes an average of 17 days. For ground-glass lesions to evolve into consolidation, it takes 7 days, pure ground-glass lesions need 8 days to evolve into ground-glass lesions. The average time for CT signs to improve is 10–15 days, and the first to improve is the crazy-paving sign and nodules; while the progression of the disease is 6–12 days, the earliest signs of progression are air bronchogram signs, bronchial wall thickening, and bronchiectasis. There is no severe patient in this study.

**Conclusion:** This study depicts the CT manifestation and evolution of COVID in non-epidemic origin areas, and provides valuable first-hand information for clinical diagnosis and judgment of patient’s disease evolution and prediction.

## Introduction

Coronavirus disease 2019 (COVID-19), an acute respiratory infectious disease in 2019–2020, is becoming more and more serious. Although the epidemic situation in China has been well controlled, the number of cases in the world is increasing, and it is still in a pandemic state. With the continuous accumulation and comprehensive understanding of disease prevention, control, diagnosis, and treatment experience, research on the disease evolution, including the risk factors for patients progressed from mild-to-severe even critical illness, risk factors for death and related early warning models, is actively processing. Imaging plays an important role in disease diagnosis, clinical decision-making, treatment, and clinical outcome evaluation. The occurrence and development of imaging is of great significance for us to understand the characteristics of the disease and to guide clinical practice. The combination of imaging findings and the nucleic acid test as a means of diagnosis program aimed to make up for missed diagnosis of early nucleic acid–negative cases, and can also be used to evaluate curative effect, and it has now become a consensus in the treatment of COVID-19. This study collects imaging and clinical data of patients with confirmed COVID-19 in 16 hospitals in southeast China outside of the epidemic area; uses big data analysis to find the early and late development; explores the correlation among imaging performance, developmental trend, and patient basic diseases; describes the characteristics and evolution of the imaging manifestations with the time of onset; displays the imaging map of new coronary pneumonia; provides imaging clues for clinical diagnosis and identification; and provides assistance in disease progression and prognostic monitoring.

## Methods

### Patients Information

The study was conducted in accordance with the Declaration of Helsinki (as revised in 2013). This retrospective multicenter study was approved by the Institutional Review Board of each participating hospital. Written informed consent was waived. A total of 693 patients came from the southeastern region of China outside of the epidemic-intensive areas, with an average age of 46 years, including 13 children; the proportion of women was 49%. They were all imported second-generation confirmed cases. This study included 1,648 chest CT examinations at 16 hospitals between January 19 and March 27, 2020. The median CT follow-up period was 10 days from symptom onset (IQR, 6–16; range, 0–58 days). All patients were recovered after treatment, and there are no clinically critically ill patients in this study.

### CT Protocol

CT examinations were performed using 16-slice to 128-slice CT scanners of different manufacturers, including SIEMENS, GE, TOSHIBA, and PHILIPS. Chest CT examinations were performed with a varying slice thickness from 1 to 5 mm. Standard lung algorithm settings were used as follows: tube voltage, 100–130 kV; tube current, 100–440 mAs; section thickness, 1–5 mm; and reconstruction matrix, 512 × 512.

### CT Image Recording Method

All CT images were read by six attending doctors with qualifications. The density of lesions was divided into pure ground-glass shadow (completely ground-glass density), ground-glass lesions (referring to ground-glass lesions containing consolidation lesions not exceeding one-half of the total volume), and consolidation lesions (completely solidified lesions or contained ground-glass opacity not exceeding one-half of the total volume). At the same time, the lobe location and distribution characteristics of the lesions were recorded: subpleural distribution pattern defined as lesions distributed within 2 cm of the subpleural area on both sides; diffuse distribution refers to the involvement of multiple lobes in both lungs; distribution along the bronchial vascular bundle pattern refers to the lesions distributed along the bronchial vascular bundle. Signs within the lesion were recorded, such as bronchogram sign, paving stone sign (shown as mosaic ground-glass shadow with thickened interlobular septum), thickened microvessels, halo and reversal halo signs, nodules, fibrous foci, and other manifestations such as pleural effusion and pericardium effusion, and old lesions. In the follow-up CT, the outcome of the above-observed lesion was recorded once more, in addition to the improvement and progress of the lesion. The form of lesion improvement is explained as the reduction of lesion area and density, including ground-glass and consolidation shadow absorption, and any kinds of density decreased and area reduced. The form of lesion progression is explained as the increase in the range and/or density of the original lesion, including pure ground-glass lesions that progress to ground-glass lesions, and ground-glass lesions that progress to consolidation lesions; disappearance of signs means improvement, and reappearance of signs means progress. The first examination was negative, and the reexamination showed that the lesion appeared and was recorded as a new lesion. The days of improvement, absorption, and progression of different density lesions and signs were recorded.

### Statistical Analysis

The differences in the frequency of the CT signs were tested with the chi-square test. The differences between the days of the onset between different CT signs were tested with the ANOVA test. The differences in the demographic data between groups were also tested with the ANOVA test. Data normality was tested with the K-W test. All statistical analyses were performed with MATLAB 2019b on windows platform. A corrected *p* value of < 0.05 was considered as statistically significant.

## Result

### Examination Information

The average time for the first CT examination was 6.5 days after the onset of the disease, and the longest CT examination was 58 days after the onset of the disease; the median number of follow-ups was two, and the maximum number of follow-ups was 10; the average follow-up interval was 5.3 days; and there were positive findings in 1,500 CT examinations.

### Basic CT Manifestations

**FIGURE 1:**
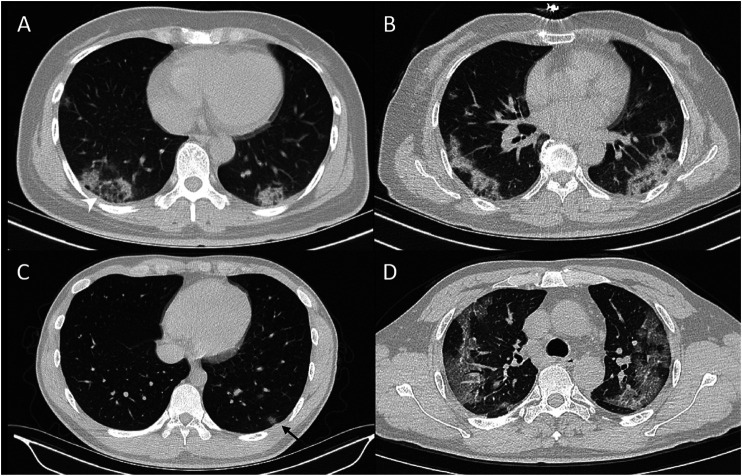
CT signs of reversed hollow sign **(A),** bilateral distribution of lesion **(B),** and nodule with hollow sign **(C)**, and crazy-paving sign **(D)**.

**FIGURE 2:**
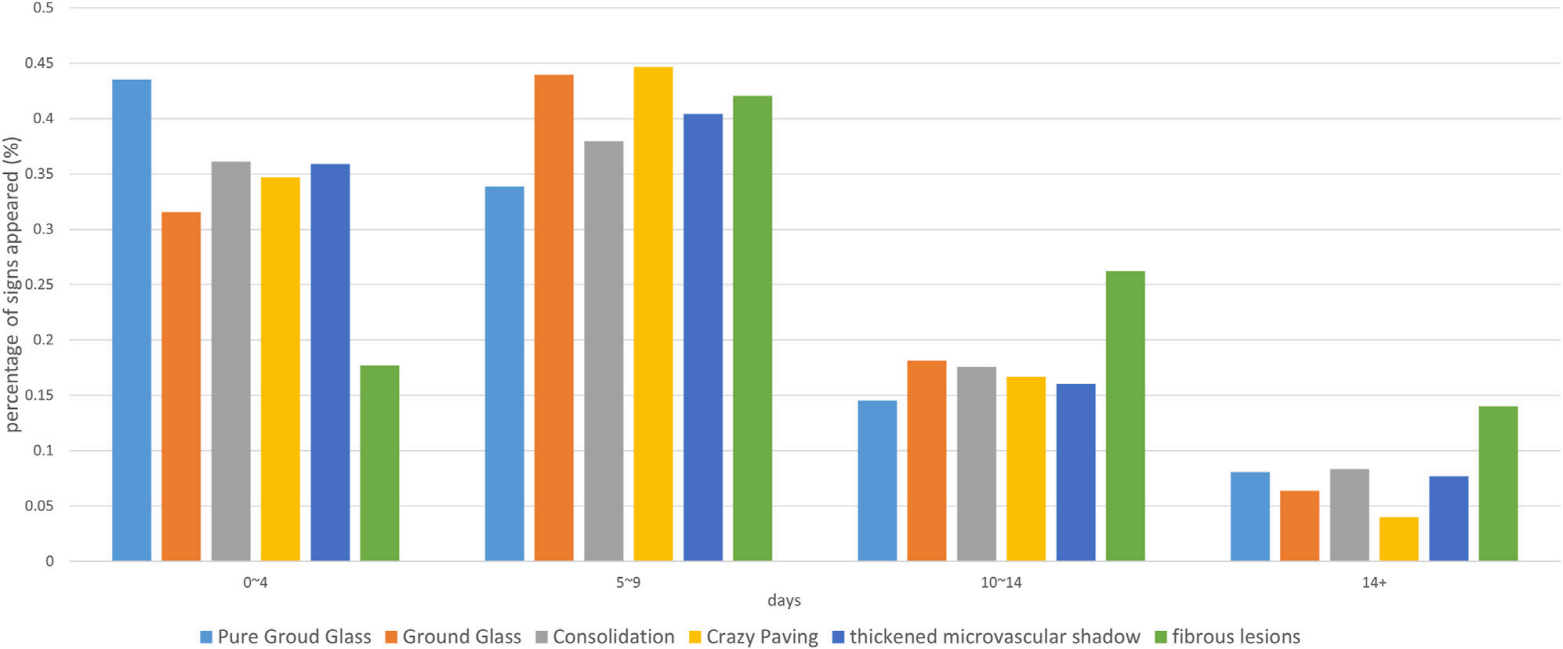
The percentage of time at each CT sign. The horizontal axis is the date of onset, and the vertical axis represents the percentage of the number of positive cases with the corresponding sign to all cases with the sign on that date.

All 1,500 CT examinations with positive signs were included, and the distribution, density, accompanying signs, and extrapulmonary manifestations were analyzed. The basic CT manifestations are as follows: 1. The distribution is mainly under the pleura, more often on the dorsal side or the lower lobes of the lungs. The lesions are mostly localized, mainly subsegmental or segmental. 2. Single or multiple nodular, patchy, or ground-glass density shadows, with or without intralobular reticular shadows, thickening of the lobular septa, and thickened microvessels and paving stone sign inside (Figures 1A–D). Consolidation was accompanied by bronchogram, internal bronchiectasis, and with or without subsegmental or lobular atelectasis, which was manifested as a fusiform, flat, or strip-shaped shadow connected to the bronchovascular bundle under the pleura (Figure 2). 3. As the course of the disease progresses and treatment measures follow-up, the ground-glass shadow or consolidation completely or partially absorbed, and was transformed into lobular atelectasis or fibrosis. Extrapulmonary manifestations including pleural effusion, pericardial effusion, and lymphadenopathy are rare.

### Frequency Analysis of Lesions

CT lesion distribution and imaging manifestation frequency analysis are shown in Table 1. The results of the chi-square test showed that the distribution of lesions was mainly subpleural (82.33%), which was significantly more than the distribution of bronchial vascular bundles or diffuse distribution, and diffuse distribution was rare. The lesions are mainly seen in the two lower lungs, and there is no obvious difference between the left and right lungs. The frequency of lesions in the middle lobe of the right lung is relatively low (51.53%); the most common manifestation is ground-glass density shadow (47.27%). The first three signs with higher frequency were fibrous lesions (42.60%), microvascular thickening (40.60%), and air bronchogram (31.87%). There was no significant difference among the three. In other accompanying signs, the paving stone sign is about 17%, and the antihalo sign is less, which is about 4%.

**TABLE 1:** The frequency of CT lesion distribution, location, intensity, and signs.

CT lesion description		Number (%)
CT lesion distribution		
Under the pleura		1,235 (82.33)^a^
Along bronchovascular bundle		573 (38.20)^b^
Diffused distribution		102 (6.80)^c^
CT lesion location		
Right upper lobe		920 (61.33)^a^
Left upper lobe		993 (66.20)^a^
Right middle lobe		773 (51.53)^b^
Right lower lobe		1,279 (85.27)^c^
Left lower lobe		1,251 (83.40)^c^
CT lesion intensity		
Ground-glass opacity		709 (47.27)^a^
Consolidation		507 (33.80)^b^
Pure ground-glass opacity		378 (25.20)^c^
CT signs of changes in the lung		
Fibrous lesions		639 (42.60)^a^
Thickened small vessel		609 (40.60)^a^
Air bronchogram		478 (31.87)^b^
Bronchial dilating and wall thickening		383 (25.53)^c^
Halo sign		309 (20.60)^c^
Crazy-paving sign		264 (17.60)^d^
Nodule		231 (15.40)^d^
Inversed halo		60 (4.00)^e^
Lobular atelectasis		29 (1.93)^f^
Pulmonary fibrosis		23 (1.53)^f^
Pulmonary emphysema		33 (2.20)^e,f^
CT signs of changes outside the lung		
Pleural effusion		21 (1.40)^a^
Pericardial effusion		3 (0.20)^b^
Enlargement of lymph nodes		32 (2.13)^b^

Frequency (%) of CT lesion distribution, location, intensity, and signs of changes in and outside the lung were shown. Within a same sub-table, the pair with the same superscript letter (a,b,c...) showed no statistically significant differences, while the pair with different superscript letter had significant differences (chi-square test, Bonferroni corrected p < 0.05).

### Analysis of Follow-Up Outcome

**FIGURE 3:**
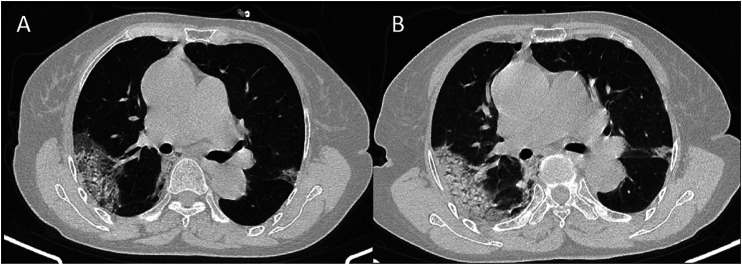
CT signs of a 74-year-old female showed subpleural distributed ground-glass opacity in 13 days **(A)** and 16 days **(B)** from onset.

**FIGURE 4:**
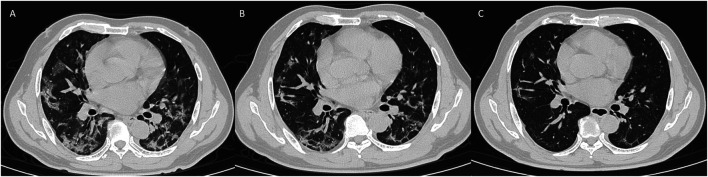
CT signs of a 47-year-old male on day 1 **(A)**, day 5 **(B),** and day 17 **(C)** showed bilateral ground-glass opacity absorbed and fibrosis formation.

The first CT examination showed significant differences in the appearance time of lesions and signs with different densities (*p = 0.014*). The average time of appearance of pure ground-glass, ground-glass lesions, and consolidation and accompanying signs including paving stone, bronchogram, thickened microvessels, nodules, halo and reversed halo signs was about 6–7 days (Table 2); among them, pure ground-glass lesions appear earliest, with the highest peak at 0–4 days (43.54% of pure ground-glass lesions appear in this period), and it took about 8 days for pure ground-glass lesions to absorb, while progress into consolidation needs 11 days (Figure 3). It takes 8 days for pure ground-glass disease to progress to ground-glass lesions. The first examination was negative, and among them, 18 patients showed original pure ground-glass lesions after 16 days of reexamination (Figure 4), 16 cases showed ground-glass lesions, and 24 cases showed consolidation lesions in about 13 days. The number of ground-glass lesions was the largest (216 cases), and the peak appeared on the sixth day. The average absorption time was 6.2 days, or it progressed to consolidation in 7 days. There were 29 cases of ground-glass lesions that evolved into pure ground-glass lesions, and the lesions took the longest time to be absorbed, which is about 17 days. Among the 29 cases, 11 patients had underlying diseases (37.93%), which was significantly higher than the other groups, including two cases of diabetes, two cases of hypertension with diabetes, two cases of hypertension, two cases of tuberculosis, two cases of spinal surgery, and one case of chronic nettle. As for age, gender, body temperature, and the proportion of neutrophils and lymphocytes, there was no significant difference between these 29 patients compared with the ground-glass absorption group.

**TABLE 2:** Comparison of time and clinical data between different outcome groups.

Trend	Lesion development	Mean days between visit	Number of cases
Recovery	Consolidation turned negative	6.37	181
	Consolidation to ground-glass opacity	10.66	71
	Consolidation to pure ground-glass opacity	13.78	23
	Ground-glass opacity turned negative	6.21	216
	Ground-glass opacity to pure ground-glass opacity	17.14	29
	Pure ground-glass opacity turned negative	7.99	78
Progressed	New pure ground-glass opacity	15.78	18
	Pure ground-glass opacity to ground glass opacity	8.73	13
	Pure ground-glass opacity to consolidation	11.07	15
	New ground-glass opacity	11.75	16
	Ground-glass opacity to consolidation	7.35	54
	New consolidation lesion	12.92	24

**FIGURE 5:**
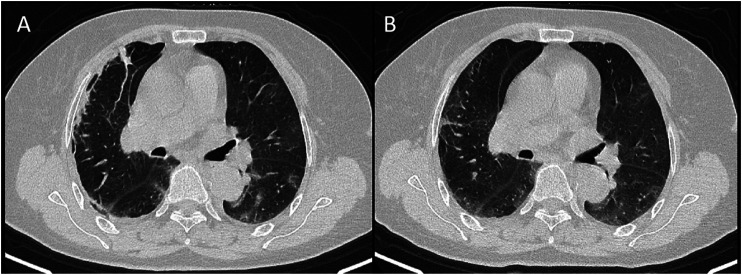
A 68-year-old female showed lobular atelectasis in the subpleural area of the right upper lobe on day 3 from onset **(A)** and disappeared on day 6 **(B)**.

The absorption time of consolidation lesions is about 6.3 days. It takes 10–14 days for consolidation to transform into ground-glass or pure ground-glass lesions. Most of the peaks of accompanying signs are on the 5th to 9th days, such as paving stone sign, microvascular thickening, and fibrous lesions (Table 3). The average time for improvement is 10–15 days. The first improvement is the paving stone sign and nodules; the time for signs to progress is 6–12 days. The earliest signs of progress are bronchogram, bronchial wall thickening, and bronchiectasis. Fibrous lesions appear less frequently in 0–4 days (17.68%), but the duration is long, and there is still 26.12% in 10–14 days, which is higher than other signs (Figure 5).

**TABLE 3:** Development of CT signs.

CT signs	From positive to negative		From negative to positive	
	Mean days between visit	Number of cases (%)	Mean days between visit	Number of cases (%)
Crazy-paving	10.14 a	62 (19.50)	10.15 b	17 (6.67)
Air bronchogram	11.32 a	79 (24.84)	6.20 b	23 (9.02)
Fibrous lesions	15.08 a	13 (4.09)	10.12 b	133 (52.15)
Bronchial dilating and wall thickening	12.88 a	56 (17.61)	9.12 b	34 (13.33)
Thickened small vessel	13.26 a	74 (23.27)	8.56 b	25 (9.80)
Nodule	10.82 a	34 (10.69)	11.48 a,b	23 (9.02)
Halo sign	11.44	54 (14.52)	9.78	16 (6.40)

## Discussion

This article summarizes and analyzes the imaging manifestation in non-epidemic areas, summarizes the image characteristics and evolution time of sporadic cases, describes the accompanying signs of the images, provides clinical diagnosis for sporadic cases and mild patients, and provides comprehensive imaging data for efficacy clinical evaluation.

### Selection of Imaging Methods

Chest CT is the first choice for screening and evaluating new coronary pneumonia (Wong et al., 2019). It is recommended to use a volumetric CT scan, the scan thickness is 5 mm (16-slice CT and above are acceptable), and the reconstruction is a thin layer of 1.0–1.5 mm. Multi-planar reconstruction (cross-sectional, sagittal, and coronal) was based on thin-slice CT. At present, the latest guidelines have not yet reached a consensus on the timing of the use of low-dose CT (LDCT) examination and the time interval for reexamination of CT. Early CT findings of some patients with new coronary pneumonia are negative, and positive findings gradually appear in the lungs as the disease progresses (Minming and Bin, 2020). We agree with opinions as follows: reports of the various CT features of COVID-19 pneumonia are an important first step in helping radiologists identify patients who may have COVID-19 pneumonia in the appropriate clinical environment. CT should be reserved for evaluation of complications of COVID-19 pneumonia or for assessment if alternative diagnoses are suspected (Raptis et al., 2020).

### CT Manifestations

#### Density Change and Evolution

Density changes include ground-glass opacity and consolidation; ground-glass opacity indicated slight increase in density as a hazy cloud-like change that does not cover the accompanying pulmonary vascular shadows in the lungs. Consolidation means a significant increase in the density of lung parenchyma, and the pulmonary blood vessels and airway walls are unclear due to the lack of alveolar gas contrast (Adam et al., 2020). The imaging manifestations depend on the pathology. The pathology of COVID-19 shows that the lungs exhibited different degrees of consolidation, mainly showing diffuse alveolar injury and exudative alveolitis. Pulmonary lesions in different areas are complex and diverse, with the new and the old lesions alternating. The formation of serous/fibrinous exudate and transparent membrane in the alveolar cavity can show consolidation of different densities in imaging. Infiltrating cells are mainly monocytes and macrophages. Type-II alveolar epithelial cell hyperplasia, alveolar septal hyperemia and edema, and some alveolar hyperinflation, forming airspace-containing cavity, can form lobular septal thickening and paving stone signs. Bronchioles and small bronchi are easy to see mucus thrombosis, pulmonary vasculitis and thrombosis, and embolism, which can explain the signs of microvascular thickening. With the developement of the disease, it can be seen that the alveolar cavity exudate is organized (meat change) and the pulmonary interstitial fibrosis, so the later stage could find the formation of fibrous bands (Minming and Bin, 2020).

The results showed that the lesions were mainly distributed under the two lower lobes, followed by the two upper lobes, and the right middle lobe was the least involved (Fan et al., 2020; Minming and Bin, 2020; Salehi et al., 2020). The frequency of ground-glass opacity is the highest, and the peak time is 0–4 days, which is consistent with many literature reports outside Hubei Province and can have no clinical symptoms (Ai et al., 2020; Xiong et al., 2020; Zhao et al., 2020). Meng et al. (2020) reported asymptomatic cases with normal laboratory findings confirmed by nucleic acid testing show CT findings as ground-glass opacity (GGO) (94.8%) with peripheral (75.9%) distribution, unilateral location (58.6%), and mostly involving one or two lobes (65.5%).

The most rapid absorption of lesion density evolution was ground glass–based lesions, with an average transformation time of 6.2 days, followed by consolidation, with an absorption time of about 6.3 days, which was also the shortest absorption time, which did not match the 3.5-day average prognosis time reported by Li et al. (2020). Shi et al. (2020) reported a case of Wuhan with pure ground-glass shadow absorption for 13 days, which the author believes is related to clinical treatment and individual differences in patients and virus virulence. It may be due to different areas of the patient. Xiong et al. (2020) reported that in Wuhan, 83% of patients, which is more than that in our observation, progressed to opacifications, consolidation, interstitial thickening, fibrous strips, and air bronchogram signs during a mean follow-up period of 4.5 and 11.6 days.

According to our result, it takes about 7–11 days for ground-glass or pure ground-glass lesions to switch to consolidation. Wang et al. (2020) reported that the ground-glass lesions progressed to a mixed type for about 12–17 days. The progression of the lesion is based on pure ground-glass opacity and develops into ground glass in turn, followed by consolidation. It is speculated that density increase is related to the increase in exudate and cells in the lung, the thickening of the interlobular septum, and the formation of fibrosis. It takes about 10–14 days for consolidation absorption to turn into ground-glass or pure ground-glass opacity; ground-glass lesions evolve into pure ground-glass and also indicate that the lesions improve absorption, but it takes as long as 17 days. The reason is that this group of patients has underlying diseases, such as hypertension, diabetes, or a history of surgery. It is speculated that the immune function of the patients may delay the absorption of the lesion. Relevant literature on clinical indicators predicting lesion progression predicted that patients with imaging progression had significantly higher frequency of chronic inflammatory manifestation than those without imaging progression (Yang et al., 2020). This may be related to the pathological basis of pulmonary edema, protein exudation, and thickening of pulmonary interstitium, which may play a role, resulting in the formation of ground-glass opacity. Among them, edema and protein exudation are absorbed faster, while the lung interstitium thickness process is relatively slow, which can explain the different results of ground-glass shadow absorption in some patients. Similarly, consolidation may be caused by multiple pathological factors. After pulmonary edema and exudation are absorbed, the density of the lesion decreases, and the interstitial thickening maintains the ground-glass density and enters a slow absorption period, which can evolve into fibrotic lesions in the later stage.

#### Accompanying Signs and Evolution

Accompanying signs include paving stone sign, microvascular thickening, bronchogram sign, halo sign, reversed halo sign, tree-in-bud sign, nodules, and traction bronchiectasis. Most signs merged in 5–9 days and accompanied with the absorption and evolution of the lesions; only fibrotic lesions appear on the 9th day. The average time for improvement is 10–15 days, and the first to get better is the paving stone sign. The progression time of signs is 6–12 days, and the relatively earlier signs are bronchogram, bronchial wall thickening, and traction bronchiectasis.

What needs to be emphasized is the paving stone sign. Some researchers believe that this sign indicates the progress of the disease; we have different opinions. The crazy-paving pattern demonstrates as thickened interlobular septa and intralobular lines with superimposition on a GGO background, resembling irregular paving stones (Cheng et al., 2020; Fan et al., 2020; Qi et al., 2020; Raptis et al., 2020; Wang et al., 2020; Ye et al., 2020). The early pathological changes of COVID-19 include pulmonary edema, protein exudation, thickening of the lung interstitium, and infiltration of multinucleated giant cells and macrophages in the alveolar cavity. Pan et al. (2020) mentioned that in 22 cases of observation, the paving stone sign appeared in 5–8 days from the symptom and continued to be absorbed after 13 days, and the lesion progression was accompanied by more paving stone signs. Salehi et al. (2020) indicated follow-up CT in the intermediate stage of disease shows an increase in the number and size of GGOs and progressive transformation of GGO into multifocal consolidative opacities, septal thickening, and development of a crazy-paving pattern, with the greatest severity of CT findings visible around day 10 after the symptom onset. There were no severe cases in this study, the paving stone sign improved in about 10 days in 20% of the patients, only 6% cases progressed, and the average progression time was also about 10 days. Relatively speaking, bronchial wall thickening appeared more frequently. In many progressive cases, thickening of the bronchial wall and microvessel signs had larger frequency and may be predictive signs of critically ill patients. The paving stone sign has been used to distinguish it from viral pneumonia. While a cluster-like pattern and bronchial wall thickening were more frequently seen in influenza pneumonia(Wang et al., 2020), we considered that the imaging manifestations of COVID and avian influenza, influenza B, and influenza A have a large overlap, and the performance is similar, and the differential diagnosis depends on clinical microbiological tests. YU reported (Yu et al., 2020) interlobular septal thickening (75%), air bronchogram (70%), and pleural effusion (40%) were more likely to manifest in patients with severe form. Wang et al. (2020) also reported heavy/critical-type COVID-19 was associated with multiple lobe involvement and the presence of consolidation, the crazy-paving sign, interlobular septal thickening, pleural thickening, and pleural effusion.

Halo sign, reversed-halo sign, tree-in-bud sign, and nodules are rare manifestations. Halo sign refers to a circle of ground-glass density shadow around the nodules or patchy opacity on CT. It was first used to describe the exudation around the focal invasive *Aspergillus* nodule. The pathological mechanism is bleeding around the lesion and so was a nonspecific sign. To COVID-19, it shows ill-defined cloud-like inflammatory exudation around the lesion, which is similar to other opacity. As for reversed-halo sign, there is a certain difference between the traditional appearance and COVID-19. The center of the reversed-halo sign showed a ground-glass density shadow, the surrounding appearance is ring or crescent, and the density of the ring in COVID-19 is lower than that of the ring in others, such as organized pneumonia. The pathological mechanism of GGO-like banding needs further explanation (Guan et al., 2020). It is reported that reversed halo sign was observed in approx. 5% of patients (Hamer et al., 2020). In the early CT images, nodules with a diameter of ≤3 cm can be solid nodules, partial solid nodules, and pure ground-glass nodules, with clear or fuzzy boundaries. As for the tree-in-bud sign, the initial lesion was a solitary sub-centimeter ground-glass nodule distributed in the boundary between the outer one-third and inner two-thirds of pulmonary lobes, along with adjacent bronchovascular bundles, and the lesion progressed over a period of 2–3 days, presenting as an enlarged nodule with a blurred margin on CT (Xia et al., 2020). Current pathological studies believe that COVID-19 mainly causes inflammatory reactions characterized by deep airway and alveolar damage. However, individual COVID-19 cases showed tree-in-bud signs, suggesting that the disease involves bronchioles (Güneyli et al., 2020).

Fibrous lesions appeared with a low frequency (17.68%) in 0–4 days, and 26.12% in 10–14 days, which is higher than that in other signs. The frequency of fibrotic lesions in the disease outcome of this study was higher, which may be due to different interpretations: some fiber streaks disappeared in the later period, and two cases of streaks were reported to be absorbed in 19 days (Jiang et al., 2020). Guan et al. (2020) reported a delay in the changes on CT and evidence of lesion absorption. During the absorption period, the patchy lung consolidations gradually absorbed and shrank, but the subpleural line was still present 22 days after admission. It is suggested that the stripe shadow displayed in the previous stage is atelectasis or segmental atelectasis. Lobular atelectasis refers to complete atelectasis or results in swelling of the lung caused by bronchial obstruction or contraction of scar tissue in the lung, which is caused by bronchiolitis. The resulting inflammation is absorbed, the lumen of the bronchioles reopens, and the lung is recruited to expand. The unabsorbable fiber strips are the real scars. Pleural effusion and enlargement of lymph nodes appear less generally. Traction bronchiectasis may appear in the consolidation area on CT or in the fibrous cord shadow.

## Research Limitations

This study mainly describes the imaging manifestations and evolution of COVID-19, and does not include the treatment and the clinical evolution of the patient. The reason is that the current treatment is basically the same, and the difference is the patient's individual response and immune status. Therefore, the image analysis of big data shows that the overall development of new coronary pneumonia has a certain degree of representativeness.

## Summary

This study describes the CT manifestation and evolution of COVID-19 in non-epidemic areas of southeast China, and provides valuable first-hand information for clinical diagnosis and judgment of disease evolution and prediction.

## Data Availability

The raw data supporting the conclusions of this article will be made available by the authors, without undue reservation.
